# Persuasive Design Techniques and App Design Recommendations to Improve Health Workforce Capability in Rural Health Professionals: What Do Users Want and How Does an App Help?

**DOI:** 10.2196/35094

**Published:** 2022-05-02

**Authors:** Sabrina Pit, Robyn Ramsden, Aaron JH Tan, Kristy Payne, James Barr, Benjamin Eames, Mike Edwards, Richard Colbran

**Affiliations:** 1 New South Wales Rural Doctors Network Hamilton Australia; 2 School of Medicine Western Sydney University Campbelltown Australia; 3 University Centre for Rural Health Faculty of Medicine and Health Sciences University of Sydney Lismore Australia

**Keywords:** health, wellness, mobile apps, persuasive strategies, behavior change, review, health workforce, capability, career, employment, rural, workforce planning, mHealth, mobile health, digital health, health professional, user experience, health application, task support, social support, dialog support

## Abstract

**Background:**

Health professionals’ perceptions of persuasive design techniques for use in technological solutions to improve health workforce capability have not been previously explored.

**Objective:**

This study aims to explore rural health professionals’ perceptions of health workforce capability and persuasive design techniques; and translate these into recommendations for designing a health workforce capability app to increase their impact and usefulness.

**Methods:**

Qualitative interviews with 13 rural health professionals were conducted. Subsequently, 32 persuasive techniques were used as a framework to deductively analyze the data. Persuasive design technique domains were Primary Task Support, Dialog Support, System Credibility Support, Social Support, and Cialdini’s Principles of Persuasion.

**Results:**

Persuasive design techniques can be applied across the factors that influence health workforce capability including health and personal qualities; competencies and skills; values, attitudes, and motivation; and factors that operate outside of work and at the team, organizational, and labor market levels. The majority of the 32 persuasive design techniques were reflected in the data from the interviews and led to recommendations as to how these could be translated into practice, with the exception of scarcity. Many suggestions and persuasive design techniques link back to the need for tailored and localized solutions such as the need for country-specific–based evidence, the wish for localized communities of practice, learning from other rural health professionals, and referral pathways to other clinicians. Participants identified how persuasive design techniques can optimize the user experience to help meet rural health professionals needs for more efficient systems to improve patient access to care, quality care, and to enable working in interprofessional team-based care. Social inclusion plays a vital role for health professionals, indicating the importance of the Social Support domain of persuasive techniques. Overall, health professionals were open to self-monitoring of their work performance and some professionals used wearables to monitor their health.

**Conclusions:**

Rural health professionals’ perceptions of health workforce capability informed which persuasive design techniques can be used to optimize the user experience of an app. These were translated into recommendations for designing a health workforce capability app to increase likelihood of adoption. This study has also contributed to the further validation of the Persuasive Systems Design model through empirically aligning elements of the model to increase persuasive system content and functionality with real-world applied data, in this case the health workforce capability of rural health professionals. Our findings confirm that these techniques can be used to develop a future prototype of an app that may assist health professionals in improving or maintaining their health workforce capability which in turn may increase recruitment and retention in rural areas. Health professionals need to be central during the design phase. Interventions are needed to provide a supportive environment to rural and remote health professionals to increase their rural health workforce capability.

## Introduction

### Background

Globally, people in rural areas are disadvantaged when it comes to seeking health care [1,2]. This manifests as poorer health outcomes and has, in part, been directly attributed to persistent recruitment and retention challenges in rural areas [3]. Additionally, high levels of burnout are reported among rural health professionals across the globe [4,5]. For example, 47.6% of rural Chinese primary health care workers reported moderate burnout and 3% severe burnout [5].

### Health Workforce Capability

There is a growing body of evidence that aims to address the barriers to, and enablers of, recruiting and retaining a rural health workforce [3,6]. For example, multiple initiatives are aimed at incentivizing work in rural areas for health professionals [7], and others demonstrate the potential effectiveness of regulatory change such as increased scope of practice for nurse practitioners [8]. Specifically, workforce capability plays a significant role in the attraction, recruitment, and maintenance of health practitioners in a rural setting [9]. Health workforce capability describes “a health professional’s overall ability to fulfil their health care role” [9]. Health workforce capability can be defined as “the intersection between individual capacity and ability to adapt to work considering the whole of health care context, including the labor market, population needs, family, schools, partner, education, and social options.” Thus, health workforce capability is a complex construct that consists of much more than just clinical competence. It is a holistic concept that considers the multidirectional interactions both internal and external to the individual practitioner [9-11]. Internally, it considers interactions between an individual practitioner’s personal and professional spheres, both of which are complicated domains that vary greatly between individuals. Externally, workforce capability takes into account the interactions between the professional and their environment, including their employer, co-workers, and social circles. For rural health professionals, it also takes into account patients, clients, and the communities they serve. Despite the clear importance of understanding capability in the context of the rural health workforce, it is an underexplored area.

### Digital Technology and Health Workforce Capability

Before the role of technology in the clinical context was forced into the limelight due to COVID-19 [12], rural health professionals globally had long been using digital solutions [12] to bridge large geographical distances. Complementary to this, recent studies have demonstrated technology’s role in supporting the workforce capability of the rural health workforce [13]. Indeed, it has been suggested that employing technology solutions in the rural context can improve the capability and retention of rural health professionals [13]. What then are the appropriate solutions to improve health workforce capability? To answer this question, the authors of this paper undertook 2 previous studies [10,11].

The first study investigated the wants and needs of the rural health workforce to improve their capability and the potential role of technology in assisting them [10]. Theories around the acceptability of information technology solutions, including the Mobile Application Rating Scale (MARS) [14], the Technology Acceptance Model (TAM) [15], and the Health Information Technology Acceptance Model (HITAM) [16] were used to address this question. The study identified 7 factors that had the greatest influence on health workforce capability: health and personal qualities; competencies and skills; values, attitudes, and motivation; and factors that operate outside of work and at the team, organizational, and labor market levels.

The second study consisted of reviewing apps in Google Play Store and technical design elements that allow the technology solution to fulfill the wants and needs of health professionals [11]. Persuasive design techniques can be used to encourage people to use a product or to take certain actions or to make certain positive decisions. Persuasion is a complex concept and can use computer-human or computer-mediated approaches. Computer-human approaches are computer driven, yet need to be programmed, so the developer has a critical role to play and has a large impact on design. Computer-mediated persuasion [17] occurs through using digital social communication tools. In the health context, this could include, for example, chats, interactive webinars, and virtual clinics where health professionals would persuade each other. Specifically, this second study investigated persuasive design techniques used to improve use of existing capability-building–related apps, and offered some basic suggestions for incorporating these techniques into a health workforce capability app to increase its persuasiveness. It used theories on persuasive design elements that influence behavioral change, including the Persuasive System Design Model (PSD Model) [17] and Cialdini’s Principles of Persuasion [18,19]. A total of 32 persuasive design techniques were studied clustered by 5 design features categories: Primary Task Support, Dialog Support, System Credibility Support, Social Support, and Cialdini’s Principles. The design techniques are further explained in Tables 1-5. While several studies have tried to understand the 2 questions independently [20,21], to our knowledge, none have examined improving health workforce capability on their own, let alone mapping the 2 together. To fill this gap, this study will map persuasive design techniques with the health workforce capability needs and perspectives of rural health professionals within the context of improving health workforce capability.

**Table 1:** Persuasive design techniques—definitions and implementation examples for developing a health workforce capability app (Domain: Primary Task Support).

Primary task support	Persuasive design technique definition^a^	Quote qualitative interviews health professionals	Implementation examples
Reduction	A system that reduces complex behavior into simple tasks helps users perform the target behavior, and it may increase the benefit-to-cost ratio of a behavior.	*Because there's no complications of magic <online course>. I just put in my password and that of course, comes up. It comes up where I left off. It tells me what I've done. It goes through everything logically. It has videos and writings and readings and downloads. And it's all so simple.* [ID number 13, female, GP^b^] *So maybe an app or any support to give you this feeling, you are confident, you didn’t leave anything behind. Maybe to add the patient’s data or - Patient X, for example, had this and that, and he needs to be checked before this date.* [ID number 2, male, GP] *There’s already heaps of computer stuff, Best Practice had a lot of various functionalities and so forth already embedded in it, for instance, the system for reminding patients’ appointments. That’s not the difficulty. The difficulty is more on - for us anyway - is more on the side of if someone needs a physio or that sort of - it’s probably case management is the thing that helps a lot, having that sort of thing and having someone refer to an allied health person easily so they can be dealt with easy, that’s where to my mind, that holistic approach, that’s, to me, the capabilities thing I guess.* [ID number 14, male, GP]	Predetermined pathways to work on health workforce capability. List of useful services. GP can book an appointment for an allied health professional with an online booking system so the patient does not have to do that, which ensures the patient is booked in and holistic care is provided.
Tunneling	Using the system to guide users through a process or experience provides opportunities to persuade along the way.	*But there are workouts that are categorised by all kinds of things, by duration, by difficulty, by area that it targets, that kind of thing, and it’s also got – if you’re a paying member you’ve also got different plans. So one of them might be a two-week plan you can follow or a six-week plan that you can follow, and you can schedule them in your calendar and they’ll send alerts saying, “Your workout’s due to start in 12 hours.* [ID number 10, female, speech pathologist] *Some of those, particularly lipid screening, that that throw out a whole range of different investigations and numbers. I'm still trying to work my way through and I don't have time, although it's not high on my to do list, to sit down and do a template to work out the flags that I have all these things. It'd be great if there was already a resource to say, here it is. If it's just two points out, tell them investigate whether they're dehydrated. If it's five points out, you need to get them to the hospital, I have my own clinical judgement that I can also use to say you need to get to the hospital. Particularly since these clinics are nurse led clinics, the buck stops with me, I'm very keen to pass the buck on if I am unsure. So, it's something that I'm having to work out by myself. As I say that, another clinician showed me a website that I can go to that’s a pathology website that I need to go through and pick out what I need. I don't always have web access, either.* [ID number 18, female, remote nurse]	An app that guides the health professional (eg, a remote nurse) through a health care protocol.
Tailoring	Information provided by the system will be more persuasive if it is tailored to the potential feeds, interests, personality, usage context, or other factors relevant to a user group.	*It’s got to be very carefully tailored.* [ID number 7, male, specialist] *Something that would update me with some areas of interest maybe, related journal stuff, so you tick literacy or whatnot and some related journal articles would pop up.* [ID number 10, female, speech pathologist]	Journal articles are tailored to health professional’s selected needs.
Personalization	A system that offers personalized content or services has a greater capability for persuasion.	*So having either an unlimited time or a flexible amount of time to complete it in is great.* [ID number 15, male, GP] *That would be something that someone else might say, “Oh, I’d like to opt out of that. Once a week is fine.* [ID number 15, male, GP]	Having a system that provides personalized offers to work on health workforce capability such as: Australian guidelines links to an online community of practice Opt-in approaches.
Self-monitoring	A system that keeps track of one’s own performance or status supports the user in achieving goals.	*You get burnout from being overworked and undervalued. And you can track that. You can track the being overworked. And you can track the value in the sense of outcomes.* [ID number 17, female, occupational therapist]	Graphs or trends and analyses showing self-rated health workforce capability level over time and time of day.
Simulation	Systems that provide simulations can persuade by enabling users to observe immediately the link between cause and effect.	*That’s very valuable to me, to see how other people actually put it into practice. So videos are quite good for that, those kind of demonstration-based things. But yeah, some summaries of research in terms of articles is also helpful.* [ID number 10, female speech pathologist]	Videos of health professionals performing certain procedures and impact on patient outcomes.
Rehearsal	A system providing means with which to rehearse a behavior can enable people to change their attitudes or behavior in the real world.	*There’s been a lot of initiative from other clinicians in my region who have wanted to use telehealth or technology for training and also professional support in regards to home visits, so things outside of the hospital environment when you’re geographically isolated.* [ID number 17, female, occupational therapist]	Simulation courses are listed and accessible to health professionals to enable rehearsal of real-world practice.

^a^Source: Oinas-Kukkonen and Harjumaa Marja [17].

^b^GP: general practitioner.

**Table 2:** Persuasive design techniques—definitions and implementation examples for developing a health workforce capability app. Domain: Dialog Support.

Dialog support	Persuasive design technique definition^a^	Quote qualitative interviews health professionals	Implementation examples
Praise	By offering praise, a system can make users more open to persuasion.	*You’ve got to...encourage good behaviours and not bad behaviours.* [ID number 7, male, pain specialist]	A system can send a positive image and message when health professionals reach part of their own set goal.
Rewards	Systems that reward target behaviors may have great persuasive powers.	*It’s got to be interactive, yeah, or else it’s just – you’ve got to give people a little encouragement, yeah.* [ID number 7, male, pain specialist]	Health care professionals get Continuing Professional Development points for completing a medical case during online training.
Reminders	If a system reminds users of their target behavior, the users will more likely achieve their goals.	*I think just a prompt can definitely make something - there’s so many strategies and things that we’re trying to remember. If someone else could remind you or prompt you, that’s one less thing to try and remember.* [ID number 6, female, occupational therapist] *Think it could be a pain in the neck [Daily test message or alert].* [ID number 13, female, GP^b^]	A reminder to take a break to recover.
Suggestion	Systems offering fitting suggestions will have greater persuasive powers.	*I guess the app can also, sort of reminders around how to stop comparing yourself to others, write down goals, celebrate your small wins. Those sorts of things. Not necessarily about, “Oh you’re doing a great job!” It’s more about, “Don’t forget to be mindful in what you’re doing but be practical at the same tim”.“ ’o, they're the“sor’s of things that really appeal to me. Yeah.* [ID number 16, female, nurse educator] *If you could put a link to something that you could go - say for example, ”You're more capable when you're relaxed. Here's a two-minute relaxation period”, whatever it is. Use two minutes of a relaxation tape.* [ID number 16, female, nurse educator]	Suggestions that the users are more capable when they are relaxed with a link to a 2-minute relaxation video.
Similarity	People are more readily persuaded through systems that remind them of themselves in some meaningful way.	*And I loved linking in to the rural webinars, conferences, that have happened. Because I’m listening to all the, I would have to say, fantastic innovations that some of these clinicians have come up with, and it is inspiration to you as a clinician. It’s like, “Oh, I can actually do that”, or, “I have the capacity to do this.* [ID number 17, female, occupational therapist]	Using medical terminology for target audience. Health professionals lead discussion groups. Health professionals demonstrate innovations that others feel can be replicated.
Liking	A system that is visually attractive for its users is likely to be more persuasive.	*And I did the preventing dementia one, and that was fantastic because it was very professional, and it was multiple modules over four weeks. They did - very professionally presented videos, fancy PowerPoints, and they would have videos that would open up where they would have question and answer sessions with the researchers.* [ID number 6, female, occupational therapist]	An app has professionally developed and visually attractive content.
Social role	If a system adopts a social role, users will more likely use it for persuasive purposes.	*We work on supporting each other that way. We just have conversations – we use Skype a lot rather than emails. Microsoft Teams I think is going to be the next step, but we actually really like that. It’s a bit like being able to pop into a room and just go, “What do you think about this? or, ‘Can you tell me what I could to do about this?’ without having to have all the formality of an email.* [ID number 9, female, physiotherapist]	An app that has communities of practices or social events. An organization supports the members if in need.

^a^Source: Oinas-Kukkonen and Harjumaa Marja [17].

^b^GP: general practitioner.

**Table 3:** Persuasive design techniques—definitions and implementation examples for developing a health workforce capability app. Domain: System Credibility Support.

System credibility support	Persuasive design technique definition^a^	Quote qualitative interviews health professionals	Implementation examples
Trustworthiness	A system that is viewed as trustworthy will have increased powers of persuasion.	*As long as there’s a privacy umbrella over it, that’s acceptable.* [ID number 7, male, pain specialist] *When I think about those things [privacy], I do feel concerned. But we’re just so enmeshed in it that it's like it's too late. So I feel like what's the point of worrying about it, it's just another thing to worry about.* [ID number 15, male, GP^b^]	App provides links to reputable websites such as Beyond Blue. Privacy statements.
Expertise	A system that is viewed as incorporating expertise will have increased powers of persuasion.	*They [the developers] need to have extensive clinical knowledge and they have to update it on a regular basis. So, Russel Harris is, of course, that's different. But, but the GPnotebook is regularly updated, so you feel you can rely on it. It uses guidelines. And they research and they make sure that they're following that.* [ID number 13, female, GP] *And also to collect clinically-actionable data that’s important data that we know makes a difference to the outcomes.* [ID number 7, male, pain specialist]	App provides extensive clinical knowledge based on latest evidence and specialist contributions.
Surface credibility	People make initial assessments of the system credibility based on a firsthand inspection.	*It doesn’t look so professional when your phone’s going, “buzz, buzz, buzz” on your desk the whole time.* [ID number 15, male, GP] *Like pharmaceutical ads...I think it’s going to the dark side really a bit.* [ID number 13, female, GP]	App is updated regularly and there are no failing links or out-of-date information. No or carefully selected commercial ads. Content portion of the app is derived from reputable sources and relevant credentials of authors are displayed prominently.
Real-world feel	A system that highlights people or organization behind its content or services will have more credibility.	*Absolutely, if I was feeling really low and I thought that someone would ring and check on me, absolutely, I think that would b e a really good thing to do. I’d be okay with that.* [ID number 9, female, physiotherapist]	App provides information about the organization or rural health professionals or both. App supports members to contact real people within the organization. Organization provides real-time support to improve health workforce capability.
Authority	A system that leverages roles of authority will have enhanced powers of persuasion.	*So I probably tap into all the pillar organisations that provide training as well.* [ID number 9, female, physiotherapist] *Yes, and I wish that we have something like this which is Australian, with Australian guidelines or something like that, because mostly of course the guidelines will be American guidelines, but I will have a quick idea and then try to find the Australian guidelines to match those.* [ID number 2, male, GP]	Australian government website reference or guidelines. Link to an official government-recognized network.
Third-party endorsements	Third-party endorsements, especially from well-known and respected sources, boost perceptions on system credibility.	*I kind of am tapping more into < Government Network> that I work with as well. So I’m part of that and that’s a really great resource, and there’s another couple of rural physios and we keep a voice for rural health in that space and also support each other.* [ID number 9, female, physiotherapist]	An official government agency has endorsed the app and this is displayed on the app.
Verifiability	Credibility perceptions will be enhanced if a system makes it easy to verify the accuracy of site content via outside sources.	*I think things that have a specific, kind of Australian – specific information that’s definitely going to be relevant to what our local health services can provide and is available locally is important as well.* [ID number 15, male, GP]	Offer access to Australian-based information. References lists and clear links to original sources.

^a^Source: Oinas-Kukkonen and Harjumaa Marja [17].

^b^GP: general practitioner.

**Table 4:** Persuasive design techniques—definitions and implementation examples for developing a health workforce capability app. Domain: Social Support.

Social support	Persuasive design technique definition^a^	Quote qualitative interviews health professionals	Implementation examples
Social learning	A person will be more motivated to perform a target behavior if (s)he can use a system to observe others performing the behavior.	*whatever we can get into in terms of progression or development and try and share that amongst ourselves and invite each other to that kind of gatherings as well.* [ID number 10, female speech pathologist] *I mean I certainly when I’m travelling to other locations for work, then I use podcasts for educational purposes, which are extremely useful while I’m driving. Now on a regular basis <major urban centre> unit run for just 30 minutes a weekly professional development, where clinicians throughout the state present on a particular topic. So you’ve got the <small town> therapist presenting, you’ve got the <small town> therapist presenting, and everyone has an opportunity to throw their ideas, have group discussion. Fantastic, and that happens at the same time every week. For professional development it’s really good, and that professional isolation, it’s an excellent tool.* [ID number 17, female, occupational therapist]	Online meetings with rural health professionals to discuss health workforce capability. Use of podcasts to listen to other health professionals when driving long distances. Display number of views for content. Allow feedback and comments on content. A discipline-specific unit (eg, occupational therapy) coordinates weekly professional development sessions, occurring at the same time, which are accessible to all clinicians across the whole state. The coordination unit can be set up in a rural area rather than a city.
Social comparison	System users will have a greater motivation to perform the target behavior if they can compare their performance with that of others.	*We do cross professional stuff too, so if there’s physios that have come across something they might forward to you.* [ID number 10, female speech pathologist] *“We just have conversations – we use Skype a lot rather than emails. Microsoft Teams I think is going to be the next step, but we actually really like that. It’s a bit like being able to pop into a room and just go, “What do you think about this? or, ‘Can you tell me what I could do about this?’ without having to have all the formality of an email.* [ID number 13, female, GP^b^]	Use chatrooms to allow for real-time discussions to compare how to improve health workforce capability. Digital badges and milestones (eg, 50 articles read).
Normative influence	A system can leverage normative influence or peer pressure to increase the likelihood that a person will adopt a target behavior.	*I feel that at the moment, I could do with a bit of help in motivation. It would be really good to discuss things with somebody, just to kind of....Almost like a careers advisor, really, in my State or somebody who would sit and say, “Well, these are the options.” Or perhaps give me new ideas.* [ID number 13, female, GP]	App provides access to career tips, and career advisors or coaches who may have a normative influence by increasing the likelihood of the health professional being motivated to work on their capability.
Social facilitation	System users are more likely to perform target behavior if they discern via the system that others are performing the behavior along with them.	*Our team communicates about training opportunities.* [ID number 9, female, physiotherapist]	Allow colleagues to share and discuss online training opportunities easily.
Cooperation	A system can motivate users to adopt a target attitude or behavior by leveraging human beings’ natural drive to co-operate.	*The benefits that I find telehealth really good for and which is actually starting to really come about is linking in to professional developments and interest groups.* [ID number 17, female, occupational therapist]	App is linking in to collaborative professional development activities and interest groups. App allows creation of community of practice. Community of practice permits creation of subgroups based on interest.
Competition	A system can motivate users to adopt a target attitude or behavior by leveraging human beings’ natural drive to compete.	*Like you can do the team things on that <exercises> and they’re much more positive in, You’ve got to do more than – to beat your whatever in the team.* [ID number 7, male, pain specialist]	Key performance indicator tracker for individual tracking of exercise or work activities or other personalized set goals that improve health workforce capability over a period (competition with self or others). Digital badges. Recognition of best practices. Mini-competitions.
Recognition	By offering public recognition for an individual or group, a system can increase the likelihood that a person/group will adopt a target behavior.	*So that recognition of skills and being valued is a huge one for senior therapists.* [ID number 17, female, occupational therapist]	Published stories of people being publicly recognized to demonstrate members are being valued for displaying capability. Digital badges.

^a^Source: Oinas-Kukkonen and Harjumaa Marja [17].

^b^GP: general practitioner.

**Table 5:** Persuasive design techniques—definitions and implementation examples for developing a health workforce capability app. Domain: Cialdini’s Principles.

Cialdini	Persuasive design technique definition^a^	Quote qualitative interviews health professionals	Implementation examples
Commitment/consistency	Are a pair of interrelated attributes in the sense that people often adhere (consistently) to their significant choices (commitments).	*And if there was an app that actually tracked what you were doing, tracked your KPIs, then that information could be funnelled back through middle management, but also the higher levels as well* [ID number 17, female, occupational therapist]	App allows for registering own KPIs^b^ (commitment) with set periods (eg, weekly, monthly [consistency]). KPIs can be communicated to supervisors and higher as requested.
Scarcity	Causes people to almost panic out of the fear that something will disappear or become unavailable, so they make an intent effort to acquire or preserve it.	—^c^	Circulation of grant opportunities with an emphasis on deadlines.
Social proof	Explains the human tendency to look around at others in society for reinforcement and direction in taking action.	*But I was thinking...that when we couldn't save the little boy that died in front of our eyes, could digitally, or through Zoom or something, could we have gotten a trauma counsellor, instead of us driving over to <rural town> , a 200 Ks. And I didn't want to drive that day, but I needed to debrief.* [ID number 3, female, remote nurse]	Shows number of members in a chat group or in a specific geographical location. Online support to assist in emergencies.
Reciprocity	Describes a human desire to make others feel appreciated by responding in ways that return good gestures.	*So it’s also very interesting just clinically – seeing what people are doing or their approaches to similar cases, so we encourage that kind of discussion a lot.* [ID number 10, female speech pathologist]	People in a community of practice help each other with clinical problems or capability-related issues.

^a^Source: Oyebode et al [20]*.*

^b^KPI: key performance indicator.

^c^No matching quote or data found.

### Objectives

This study aims to:

1. explore rural health professionals’ perceptions of health workforce capability and persuasive design techniques;
2. translate these into recommendations for designing a health workforce capability app to increase their impact and usefulness.

## Methods

### Data Collection

A qualitative analysis of interview data used in the 2 previous studies was conducted to evaluate how persuasive design techniques can be used to build a health workforce capability app. As outlined in Ramsden et al [10], 13 rural health professionals were interviewed about their understanding of health workforce capability, their perceived needs to improve health workforce capability, and how technological solutions can assist in improving rural health workforce capability and intentions to remain in rural practice.

Interview questions were informed by the work of Anderson and co-workers [22] and Jeffrey and colleagues [23] in terms of focusing on technology acceptance and behavior change. Persuasive strategy questions were informed by the work of Oyebode and co-workers [20] as described above.

Recruitment occurred via the telephone, in person, or through the Rural Health Pro newsletter. Rural Health Pro is a digital platform that links health professionals and organizations interested in rural health [24]. A plain language statement was emailed to participants expressing interest. An interview time was established after consent. Telephone interviews were conducted by RR and SP, digitally recorded, and transcribed. Transcripts were not returned to participants for comment, verification, or correction. Upon transcription, identifying information was removed. The transcribed interviews were managed in MS Word. Both RR and SP have extensive experience in conducting qualitative interviews. SP has lived rurally since 2005 and worked in rural health research since 2006. RR has worked in rural health since 2012. These experiences have shaped the interviewers desire to improve rural health workforce.

Participants included general practitioners (GPs), a pain specialist, nurses, and allied health practitioners. The group comprised 4 males and 9 females. Age ranged between 39 and 65 years, with an average age of 51 years. Interviews took on average 46 minutes (range 29-98 minutes). The interviewers had no previous existing relationship with the participants. RR and SP undertook reflexivity exercises during the data collection to ensure rigor, in addition to the checks during analysis listed below. RR and SP recognized that they have an interest in improving rural health workforce capability using technology. Both ethical and practical issues that arose during the interviews were discussed between the 2 interviewers to ensure alignment and rigor when conducting the interviews.

### Analyses

Our systematic review of persuasive apps that are related to health workforce capability generated examples of how the various techniques can be used in practice to develop a health workforce capability app. Subsequently, the 32 persuasive techniques [11,17-19] were used as a framework to deductively analyze the data. Descriptions of the 32 persuasive design techniques are listed in Tables 1-5.

Verbatim transcripts were coded manually. The first 2 recordings were coded separately by 2 authors (KP and RR). The remaining 11 interviews were coded primarily by authors KP and SP. RR and AT were involved in coding transcripts and reaching consensus. SP and KP subsequently used the coded data to analyze and identify quotes to support the development of suggestions for how persuasive design techniques can be used to build a health workforce capability digital tool. The findings were discussed and checked by RR and AT to ensure validity of the data. JB provided specific feedback on user design and discussions were held to further shape the data interpretation.

### Ethical Approval

The study was approved by the North Coast NNSWLHD Human Research Ethics Committee (2020/ETH03020).

## Results

Tables 1-5 show how health professionals’ needs and suggestions are reflected in the 32 persuasive design techniques that can be used when building a health workforce capability online support tool.

The results are displayed by the main domains in persuasive design techniques: Primary Task Support, Dialog Support, System Credibility Support, Social Support, and Cialdini’s Principles of Persuasion. The researchers analyzed the qualitative data to identify how health professionals’ perspectives could be translated into recommendations for designing a health workforce capability app to increase their persuasiveness. The majority of the 32 persuasive design techniques were reflected in the quotes from the interviews and led the researchers to a recommendation as to how these could be translated into practice, with the exception of competition and scarcity.

Primary Task Support techniques, for example, may be utilized to guide health professionals through a clinical protocol or professional development activity by reducing complex behaviors into simple tasks, tailoring evidence-based information to needs, and personalizing content (eg, Australian guidelines). Further, techniques such as simulation would enable health professionals to observe other clinicians performing procedures and rehearse behaviors themselves, accommodating health professionals’ ideas of how they would like to use an app to build capability.

Health professional perspectives also highlighted the importance of credibility for app design. For example, under the domain System Credibility Support (Table 3) a GP mentioned “Like pharmaceutical ads...I think it’s going to the dark side really a bit” [ID number 13, female, GP], suggesting that consideration should be given to developing criteria about which type of ads would be acceptable. Simultaneously, given the importance placed by the participants on addressing various social needs to improve health workforce capability, designers would need to consider eligibility criteria for joining online communities to safeguard the quality of online community members (System Credibility Support).

Giving feedback to support health professionals to move toward their goals, such as rewards and praise in the form of continuing professional development points or encouragement for goal-directed behaviors, is a suggested design feature. Further, reminders about target behavior, such as taking a break, and suggestions related to capability-supporting behaviors that are suitable or appropriate to health professionals were examples provided that align with Dialog Support techniques.

Further analyses revealed that health professional needs and digital solutions mapped against persuasive techniques broadly align with the factors affecting health workforce capability. The analyses are summarized in Table 6. Based on health practitioners’ insights and suggestions, practical recommendations have also been presented in Table 6. It is noted that the findings are not necessarily mutually exclusive given the complexity. For example, depending on the level and governance, the recommendation about communities of practices on team level can also be viewed as Primary Task Support and Social Support if, for example, a national or state body would manage the communities of practice. The recommendation relating to organizational level refers to the fact that referrals and closed-loop communication are professional activities that form part of the practitioner’s primary scope of work. Thus, this function would be simplifying a process that already takes place and falls within the definition of reduction. The design techniques appear acceptable to rural health professionals and can be incorporated into future apps that focus on improving or maintaining health workforce capability across several areas.

**Table 6:** Factors influencing health workforce capability mapped against persuasive design techniques (design feature category).

Factors influencing health workforce capability and persuasive design technique			Persuasive design feature domain		Recommendations provided by participants that can be linked with health workforce capability factors
**Health and personal qualities**					
	Self-monitoring	Primary Task Support		A reminder on an app to take a break to recover.	
	Reminder	Dialog Support		Improved fitness through an exercise monitoring and scheduling app.	
**Competencies and skills**					
	Expertise	System Credibility Support		An app that provides extensive clinical knowledge based on latest evidence and specialist contributions. Clinical competence building through online education.	
**Values, attitudes, and motivation**					
	Surface credibility	System Credibility Support		Increased credibility by not displaying pharmaceutical advertisements on apps.	
	Recognition Social learning Social facilitation	Social Support		Published stories of people being publicly recognized for work they have done to demonstrate members are being valued.	
**Factors outside of work**					
	Social comparison Social learning	Social Support		Using chatrooms to allow for real-time discussions and to see other rural health professionals taking a holiday.	
**Team level**					
	Reciprocity	Cialdini’s principles		Health professionals participating in a community of practice and helping each other with clinical problems or capability-related issues.	
**Organizational level**					
	Reduction	Primary Task Support		GPs^a^ being able to book patient appointments for allied health professionals online and that which link back to the GP so both parties receive reports. This could potentially work both ways. Allied health professionals being able to book appointments with the GP for their patients.	
**Labor market**					
	Tailoring	Primary Task Support		Job vacancies for rural health professionals are tailored to health care professionals’ interest and discipline.	

^a^GP: general practitioner.

## Discussion

### Principal Findings

A qualitative analysis of semistructured interviews with rural health professionals was undertaken to investigate the alignment of user perceptions of health workforce capability with persuasive design techniques. These findings were then translated into recommendations for designing a health workforce capability app to increase their impact and usefulness (Tables 1-6). The authors found that the persuasive design techniques can be applied across the factors that influence health workforce capability including health and personal qualities, competencies and skills, values, attitudes, and motivation, as well as factors that operate outside of work, and at the team, organizational, and labor market levels. There is alignment between the needs of health professionals and persuasive design techniques. The health professionals’ interviews clearly identified the persuasive design techniques that were appealing. Many suggestions link back to the need for credible, tailored, and localized solutions such as having Australian-based evidence, localized communities of practice, access to learning from other rural health professionals, and referral pathways to other clinicians.

It was also clear that rural health professionals in our study aspired to have more efficient systems to improve patient access to care, quality care, and to enable working in interprofessional team-based care. Persuasive design techniques that can assist here are, for example, tailoring, reduction, social roles of digital technology, and authority. These techniques can influence team and organizational factors to improve health workforce capability. Indeed, the need for team-based care is an old adage, and barriers and facilitators to this have been explored over time [25]. Notably, our previous work [10] demonstrated that COVID-19 has accelerated the capability and willingness for team-based care arrangements in primary and remote care through using digital solutions. This study has clear recommendations as to how persuasive design techniques can be used in digital solutions to further improve patient access, quality care, and promote team-based care. For example, one GP recommended that GPs should be able to book an appointment with a local allied health professional with an online booking system (Primary Task Support) so the patient does not have to do that. This ensures the patient is booked in (tunneling) and reduces the risk of the patient not following up with their allied health appointment, thereby enhancing the quality of care as well as the GPs own health workforce capability. Ways in which this system may improve a GPs and allied health professional’s workforce capability are reduced red tape and less inconvenience in making referrals; improved close loop communication that reduces the workload for the GP and the allied health professional; better outcomes for patient that leads to further reduction in the future workload; positive feedback to GPs and allied health professionals (recognition); and improved job satisfaction for both allied health professionals and GPs as closer relationships may be developed overtime.

Overall, health professionals were open to self-monitoring of their work performance and some already regularly use wearables to monitor their health. Although using wearables to measure work performance and stress levels at work has potential [26], it is still in its infancy. A 2019 randomized controlled trial showed that using wearables to improve emergency physicians’ well-being through monitoring their pulse rate while at work was feasible but not recommended [27]. The authors found that the biosensor could not provide reliable estimates of metrics of interest in their study context. However, Ferdous and colleagues [28] found that patterns of smartphone app usage were correlated with stress levels in work environments and thus recommended that these could be used to measure stress at work. Alhasani et al [29] presented the possibility of an app that creates behavioral data, based on self-report and sensors, that can be analyzed in real-time to predict users’ needs and provide tailored interventions for the user. In the case of rural health workforce capability, if a health professional’s heart rate variability indicates high stress levels derived from a sensor, the app can recommend a predetermined meditation session. The behavioral data can also be merged with self-reports generated by in-app journals or self-report. Synthesizing and interpreting this information using machine learning could improve the accuracy of predictions and hence provide more targeted and personalized recommendations for the user.

Social inclusiveness is a known contributing factor for improved health workforce capability [10]. Therefore, computer-mediated persuasion [17] using digital social communication tools that have a real-world feel and demonstrate that there are real people and experts (surface credibility) behind a digital tool or content is important. Health professionals will be more likely to engage with a digital system if they know that real people and respected experts are involved. In a study of nurses, Mayer and colleagues [30] found that approximately half of the nurses used apps for their work and stressed the importance of apps being validated by credible bodies before they can be used in practice. Expert design, content, and involvement will lead to greater system credibility and also improved social connections and support channels for rural health professionals. This stresses the importance of policies to ensure high-quality membership of digital communities and, thus, system credibility (persuasive design technique) to guarantee impact and engagement with the tool. Ultimately, the quality of the online membership can have an impact on usage and engagement of health workforce capability apps. Therefore, factors to consider when developing an app are closed or open membership; verification of credentials; potential publication of credentials; role and credentials of moderators; and rules of participation.

### Validation of Persuasive Systems Design Model and Persuasive Design Theories

Our work has contributed to the further validation of the Persuasive Systems Design model [17] through empirically aligning elements of the model to increase persuasive system content and functionality with real-world applied data, in this case the health workforce capability of rural health professionals. We analyzed the *use context* through identifying problem domain–dependent features which are the factors that influence health workforce capability [10]. We also analyzed the *user context* that are the user-dependent features such as goals or motivations of health professionals [10]. Subsequently, we analyzed the *technology context* through identifying the technology-dependent features for health workforce capability–related apps [11]. In this paper, we then described example software apps and implementations. Some of these examples are relatively novel in addressing health workforce capability needs such as geolocation of health professionals in a rural area to reduce social isolation [10] and some are already used in varying capability building–related apps such as easy access to clinical guidelines to increase medical performance [31] in apps such as UptoDate [11] or online communities such as Rural Health Pro [24]. Rural Health Pro is a digital platform that connects people and organizations who care about keeping rural communities healthy. Notably, we found that sometimes comments made by health professionals could apply to several persuasive design techniques. This is confirmed by feedback from user experience designers that, for example, tailoring and personalization are often the same in practice.

### Study Strengths and Limitations

Overall, the alignment between the needs of health professionals and persuasive design techniques to improve health workforce capability through optimizing the user experience means that we can support rural health professionals which may lead to greater retention [9,11,13]. The above findings are important as they confirm that these techniques can be used to develop a future prototype that may assist health professionals in improving or maintaining their health workforce capability which in turn may increase recruitment and retention in rural areas. However, we stress that it is unlikely that a single app could cover all persuasive design techniques explored in this paper, nor do we imply a health workforce capability app should do so. For example, if we built a health workforce capability app for GPs, it likely would not have the ability to book appointments for allied health professionals as that may not make sense from a product or commercial perspective to bundle and market those features together. Additionally, a system itself needs to provide enough value and solve a problem for health professionals, regardless of the number of persuasive techniques built into the app. A very useful system without any or a limited number of persuasive techniques could still improve health professional capability. Persuasive design techniques are only one element to consider in the development process of a health workforce capability app. For example, usability heuristics are useful to develop an easy-to-use interface, marketing campaigns may be necessary to promote uptake of a health workforce capability app, and service blueprinting can be used to guide the whole process.

### Ethical Considerations

It is important to consider the ethic factors in using persuasive strategies. Jacobs [32] explored the use and ethical concerns of persuasive technology for vulnerable people. While health professionals are not considered vulnerable overall, they can be vulnerable when working in isolation for long periods and this may be exacerbated by the COVID-19 pandemic and other natural disasters such as bushfires, droughts, and floods. Their strong sense of responsibility and commitment to their patients and community make health professionals potentially more vulnerable over time as their energy levels and resources are depleted [33]. However, the point of using persuasive design techniques in digital solutions is to make health professionals feel more capable when they are feeling overstretched or underconfident. The implication is that thorough consideration of, and trialing of, appropriate strategies is important.

Vulnerability can be caused by intrinsic factors (within a person) or can be caused by external factors that are situational specific [32]. An example of situational vulnerability is that an app may be designed for support but reduces the health professional’s privacy, and increases feelings of being watched. It may also increase their feelings of anxiety about the potential for negative consequences if their capability goes down, and they could potentially start to feel powerless. The ethical concept of autonomy may be violated in this example.

The design should ensure that the user, in this case, the health professional, can easily retract their consent to take part to avoid coercion [32]. This should include the ability to withdraw any data already submitted or collected about them. This will have the secondary function of improving engagement as the participants are assured of the ability to cease their involvement at any time. For example, a rural health professional when highly capable may be willing to partake in an app that measures their capability; however, if they feel less capable for any reason, it may well be that they become increasingly vulnerable. Completing a rating on a regular basis can make people aware of their shortcomings and have unintended consequences [32], such as exacerbated feelings of incompetence or reinforcing unconscious incompetence.

Jacobs [32] recommends that to safeguard against unintended consequences, technology designers should understand the experiences, interests, and needs of prospective users through inclusion of vulnerable populations at every stage of the design process. Our study described here has done that and is the first step in the co-design process. Designers need to ensure they understand the values, needs, and interests that are important to the users. A second safeguard is to take into account real-life contexts, which can reduce unintended negative consequences [32]. The latter issue demonstrates the importance of considering a digital tool as part of an integrated approach that includes a real-life support component. Finally, there must be a clear and continuous feedback mechanism where users can identify and raise issues of concern for the developers to remedy.

Organizations developing or managing apps or online communities would need to ensure high-quality online membership. Ethical principles, membership criteria, and policies will need to be developed to ensure mechanisms are in place to ensure members or users genuinely represent the purpose and values of the community.

To design a digital health workforce capability solution, it is important to select and apply an ethical framework that fits the health workforce capability needs and assists in carefully weighing up risks and benefits. Many ethical frameworks exist in the medical research field. They can be disease specific or goal specific such as an ethical framework for COVID-19 contact tracing [34] or more general such as the work conducted by Tokgöz and co-workers [35] who developed an ethical framework for health and medical apps based on a systematic review and expert interviews. However, it is ultimately recommended to use an ethical framework that aligns with international standards. Several international standards are available and selection of a standard will depend on the context of the app such as ‘ISO/TS 82304-2:2021 Health software — Part 2: Health and wellness apps — Quality and reliability’ [36] or ‘the ISO/TS 17033:2019 Ethical claims and supporting information — Principles and requirements’ [37]. The latter can be used when specific standards are not available or can complement existing standards. It covers principles and requirements for developing and declaring ethical claims.

Given that rural health professionals are often subject to burn out due to isolation, high stress, long working hours, lack of local staff, and limited resources, being able to provide a technology solution with persuasive strategies to boost capability is a positive aspect.

### Conclusions

Rural health professionals’ perceptions of health workforce capability informed which persuasive design techniques can be used to optimize the user experience of an app. These were translated into recommendations for designing a health workforce capability app to increase likelihood of adoption. This study has also contributed to the further validation of the Persuasive Systems Design model through empirically aligning elements of the model to increase persuasive system content and functionality with real-world applied data, in this case the health workforce capability of rural health professionals. Our findings confirm that these techniques can be used to develop a future prototype of an app that may assist health professionals in improving or maintaining their health workforce capability, which in turn may increase recruitment and retention in rural areas. Health professionals need to be central during the design phase. Interventions are needed to provide a supportive environment to rural and remote health professionals to increase their rural health workforce capability.

## Abbreviations

GP: general practitioner
HITAM: Health Information Technology Acceptance Model
KPI: key performance indicator
MARS: Mobile Application Rating Scale
PSD: Persuasive System Design Model
TAM: Technology Acceptance Model
